# The Effects of Botulinum Toxin on Vascular Diameter: A Preliminary Report

**DOI:** 10.1111/jocd.70113

**Published:** 2025-03-12

**Authors:** Shahriar Nazari, Nima Hadadian, Foroohe Bayat, Mohammad Reza Pourani, Cristina Muñoz‐Gonzalez, Nabil Fakih‐Gomez

**Affiliations:** ^1^ Department of ENT and Head and Neck Surgery BMI Hospital Tehran Iran; ^2^ School of Medicine Tabriz University of Medical Sciences Tabriz Iran; ^3^ School of Medicine Ahvaz Jondishapur University of Medical Sciences Ahvaz Iran; ^4^ Department of Dermatology Shohada‐e Tajrish Hospital, Shahid Beheshti University of Medical Sciences Tehran Iran; ^5^ Department of Facial Plastic & Cranio‐Maxillo‐Facial Surgery Fakih Hospital Khaizaran Lebanon; ^6^ Department of Surgery University of Salamanca Salamanca Spain

**Keywords:** botulinum toxin, cosmetic dermatology, cosmetic procedure, vascular

## 1 Introduction

Botulinum toxin (BoNT) is an exotoxin generated by the Gram‐positive anaerobic bacterium *Clostridium botulinum* . Among the seven serotypes of this toxin (A, B, C, D, E, F, and G), serotypes A and B are utilized in clinical practice [1]. BoNT functions as a neurotoxin by binding to presynaptic neurons, thereby preventing the release of acetylcholine at the neuromuscular junction. This action results in the paralysis of striated muscles for a period of 3–6 months. Overall, BoNT is considered safe, with mild and transient side effects [2].

BoNT is employed across various medical specialties, including neurology (e.g., torticollis, dystonic tics, spastic dystonia), ophthalmology (e.g., strabismus, blepharospasms, corneal astigmatism), dermatology (e.g., hyperhidrosis, rosacea, scar prevention), urology, and gynecology [3]. In cosmetic medicine, BoNT is indicated for treating facial wrinkles such as crow's feet, glabellar lines, perioral lines, forehead lines, gummy smiles, and masseter hypertrophy. Notably, emerging applications include the treatment of parotid gland hypertrophy for aesthetic purposes [4]. Additionally, recent therapeutic uses of BoNT encompass eccrine hidrocystomas, enlarged pores, keloids, hypertrophic scars, hidradenitis suppurativa, salivary gland hypertrophy, and hair regrowth [5].

Interestingly, in animal models, BoNT administration has been shown to increase the survival of various types of flaps [6]. Furthermore, BoNT induces vasodilation and reduces thrombosis in arteries and veins [6]. Several studies have also reported the efficacy of BoNT in treating Raynaud's phenomenon (RP) [7].

This case series provides a comprehensive evaluation of the effects of BoNT on vascular diameter in a selected cohort of five patients. The investigation aims to explore the immediate effects on vascular diameter, delving into the broader implications for therapeutic strategies involving BoNT in vascular ischemia.

## 2 Materials and Methods

The study included patients without cognitive impairments or psychiatric pathologies, following strict inclusion and exclusion criteria. Patients were excluded if they had known allergies to botulinum toxin type A (BoNT‐A), human albumin, or presented with generalized muscular diseases, infections, or inflammation in the treatment area. Additional exclusion factors included conditions affecting the stomatognathic system, such as trigeminal neuralgia, signs of ptosis, or any pathological decrease in muscle activity. Those who had undergone recent upper‐third facial surgeries or aesthetic treatments with BoNT‐A within the past 6 months were also excluded. The study adhered to the guidelines of the Declaration of Helsinki (1996) and good clinical practice.

At baseline, we measured the diameter and velocity of the supratrochlear arteries using the Clarius L20 (Clarius Mobile Health, Canada) handheld high‐frequency ultrasound device (HD, 8–20 MHz). Eight units of BoNT‐A (Dyston, abobotulinum toxin A; Atra Zist Aray Biopharmaceutical, Iran) were then administered intramuscularly into the frontalis muscle adjacent to the highest point of the left supratrochlear artery near the hairline. As a control, normal saline was injected on the contralateral side, adjacent to the right supratrochlear artery at the corresponding point. After 30 min, the diameter and velocity of both supratrochlear arteries were reassessed by a blinded operator. All patients included in this study provided written informed consent.

Quantitative and qualitative data were reported as mean ± standard deviation and frequency (percent), respectively. To assess the normality of the data, we utilized the Kolmogorov–Smirnov test. We used the Wilcoxon and Mann–Whitney *U* tests to compare data before and after the procedures. The Wilcoxon signed‐rank test was used due to the small sample size and potential non‐normality in subgroups. A *p* value of less than 0.05 was considered statistically significant. All statistical analyses were performed using SPSS (version 26).

## 3 Results

We initially assessed seven patients for BoNT‐A administration, excluding two due to recent BoNT injections. Five patients (three females and two males; mean age: 31.40 ± 6.80 years, range: 17 years) with no recent history of BoNT‐A use were included in the evaluation. The diameter of the supratrochlear artery was similar on both sides: 0.87 ± 0.38 mm on the left and 1.07 ± 0.31 mm on the right (*p* = 0.55).

The diameter of the supratrochlear artery increased to 1.69 ± 0.27 mm (range: 0.72 mm) following BoNT‐A injection, which was significantly larger compared to the baseline measurement of 0.87 ± 0.38 mm (range: 0.88 mm; *p* = 0.043). In contrast, there was no notable change in the diameter of the supratrochlear artery with normal saline administration, with measurements of 1.07 ± 0.31 mm (range: 0.75 mm) before and 1.01 ± 0.28 mm (range: 0.65 mm) after the injection (*p* = 0.5). The supratrochlear artery diameter difference between the BoNT‐A and normal saline sides, both at baseline and postinjection, was 0.88 ± 0.20 mm. Additionally, Cohen's *d* was 4.4, indicating a substantial effect size.

**FIGURE 1:**
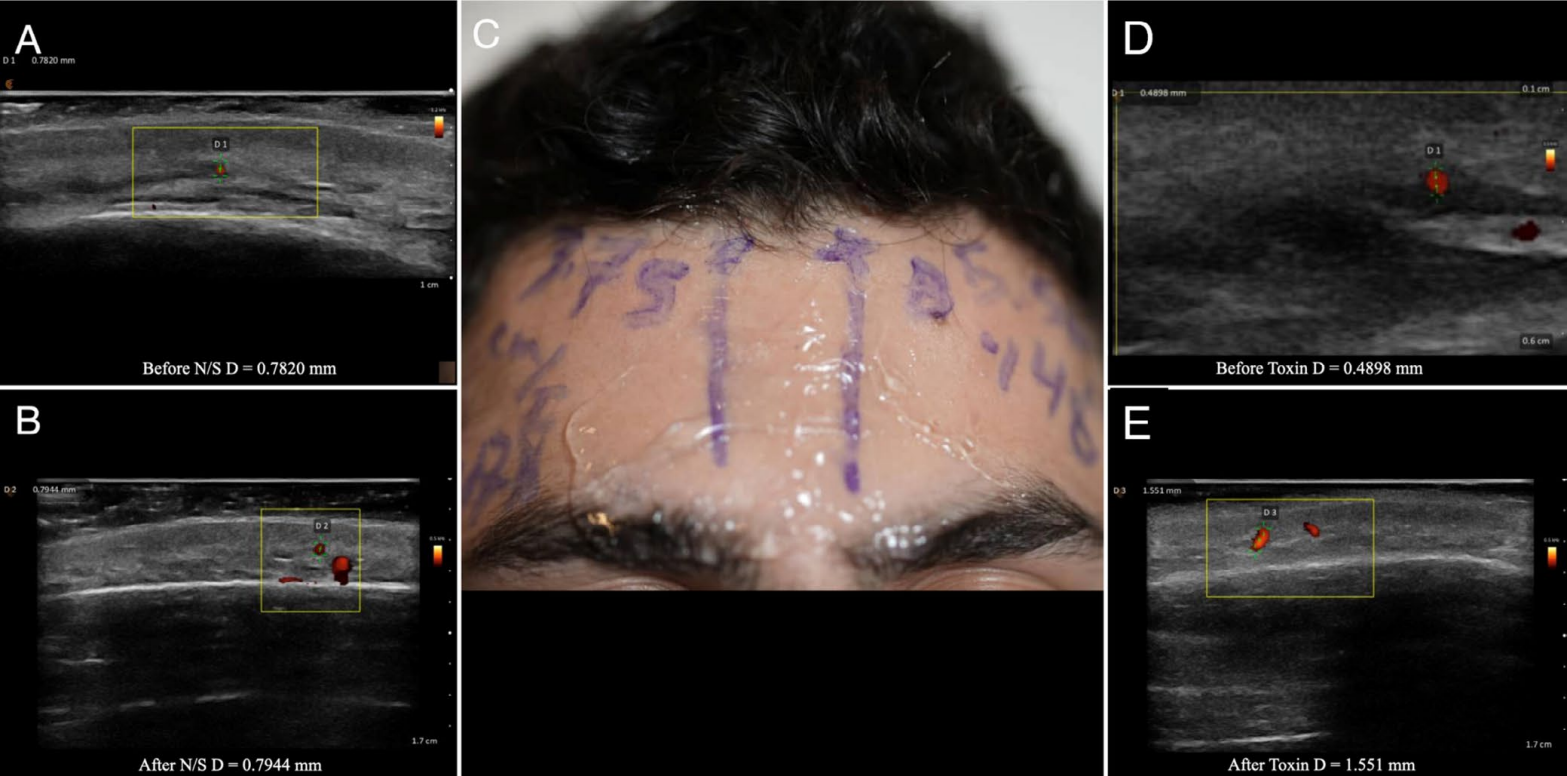
Split‐face study of a 25‐year‐old male patient, with normal saline injected into the right artery and 8 IU of botulinum toxin injected into the left supratrochlear artery. (A) Ultrasound image of the right supratrochlear artery before injection. (B) Ultrasound image of the right supratrochlear artery after injection of normal saline. (C) Marked supratrochlear arteries on the split face of the patient, with accompanying measurements. (D) Ultrasound image of the left supratrochlear artery before injection. (E) Ultrasound image of the left supratrochlear artery after injection of botulinum toxin.

**FIGURE 2:**
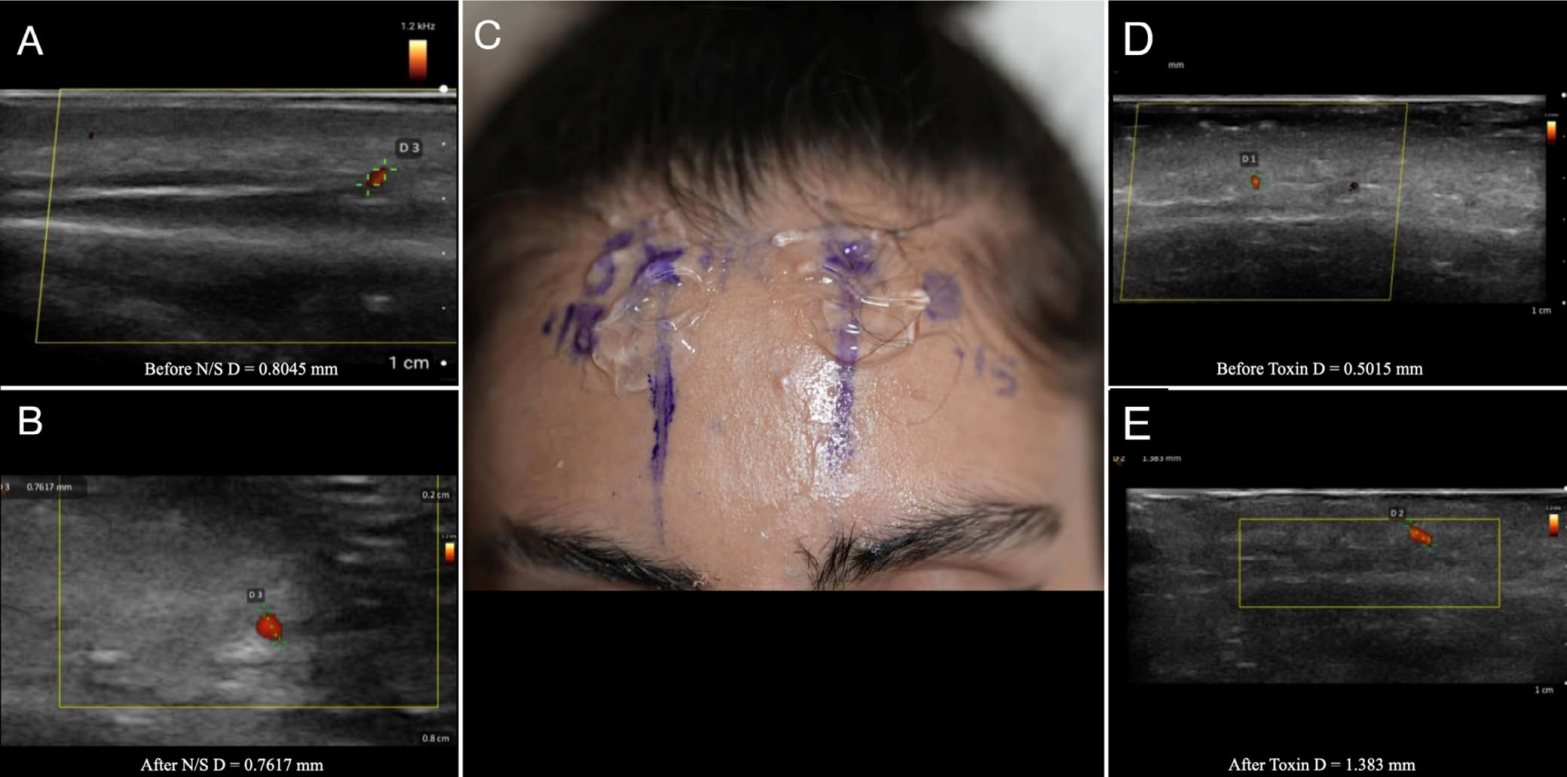
Split‐face study of a 26‐year‐old female patient, with normal saline injected into the right artery and 8 IU of botulinum toxin injected into the left supratrochlear artery. (A) Ultrasound image of the right supratrochlear artery before injection. (B) Ultrasound image of the right supratrochlear artery after injection of normal saline. (C) Marked supratrochlear arteries on the split face of the patient, with accompanying measurements. (D) Ultrasound image of the left supratrochlear artery before injection. (E) Ultrasound image of the left supratrochlear artery after injection of botulinum toxin.

**FIGURE 3:**
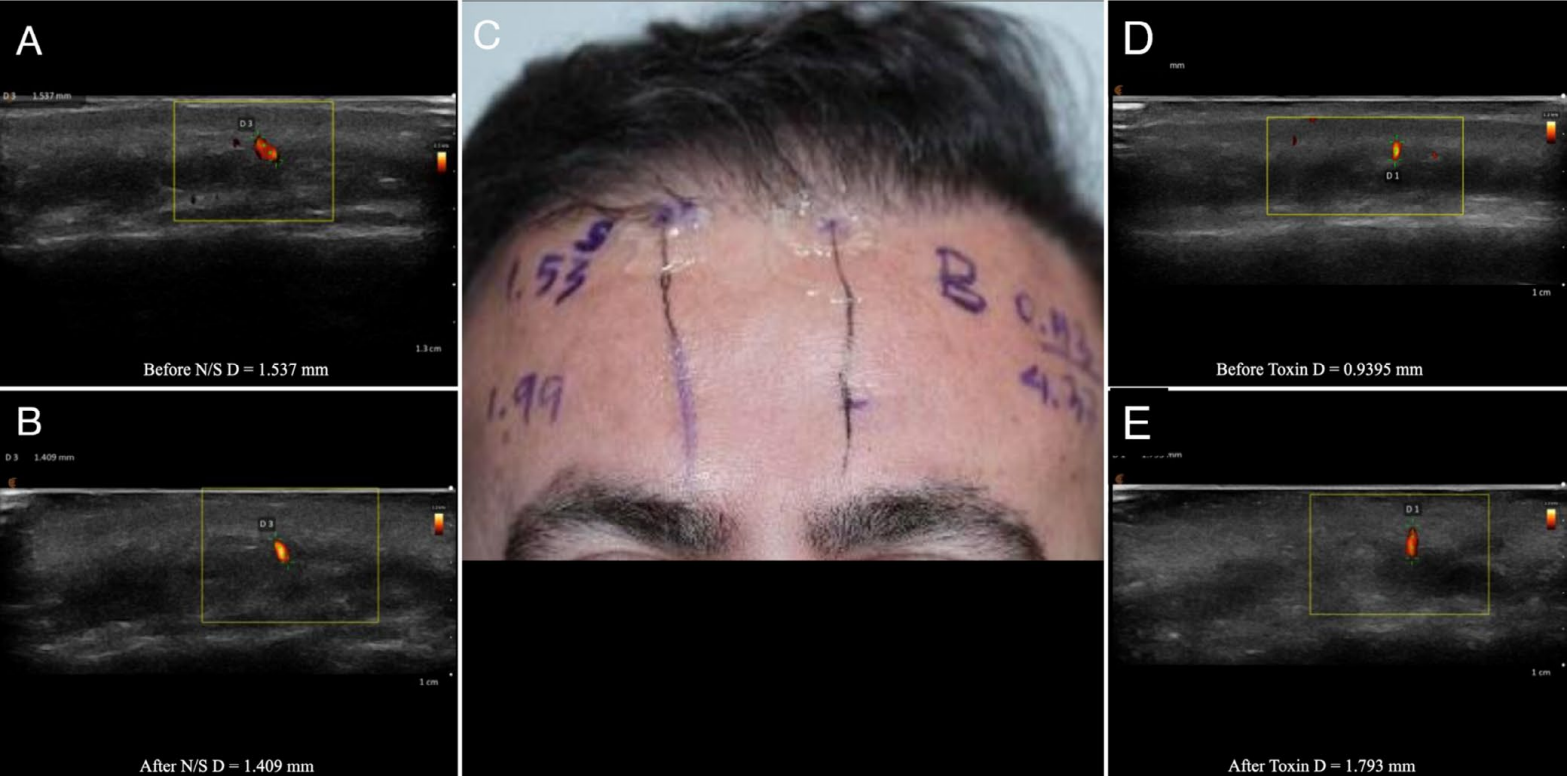
Split‐face study of a 42‐year‐old male patient, with normal saline injected into the right artery and 8 IU of botulinum toxin injected into the left supratrochlear artery. (A) Ultrasound image of the right supratrochlear artery before injection. (B) Ultrasound image of the right supratrochlear artery after injection of normal saline. (C) Marked supratrochlear arteries on the split face of the patient, with accompanying measurements. (D) Ultrasound image of the left supratrochlear artery before injection. (E) Ultrasound image of the left supratrochlear artery after injection of botulinum toxin.

**FIGURE 4:**
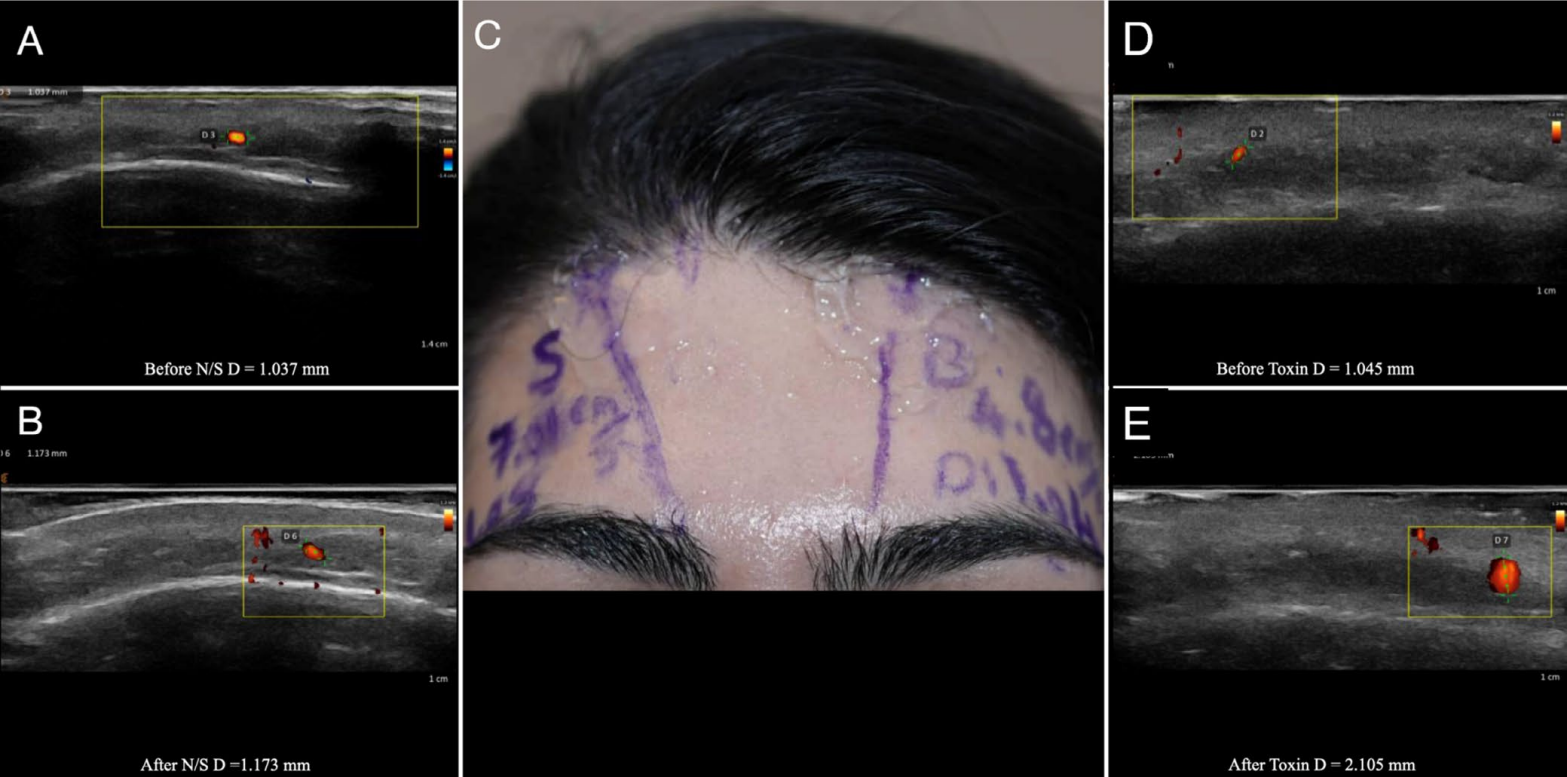
Split‐face study of a 31‐year‐old female patient, with normal saline injected into the right artery and 8 IU of botulinum toxin injected into the left supratrochlear artery. (A) Ultrasound image of the right supratrochlear artery before injection. (B) Ultrasound image of the right supratrochlear artery after injection of normal saline. (C) Marked supratrochlear arteries on the split face of the patient, with accompanying measurements. (D) Ultrasound image of the left supratrochlear artery before injection. (E) Ultrasound image of the left supratrochlear artery after injection of botulinum toxin.

**FIGURE 5:**
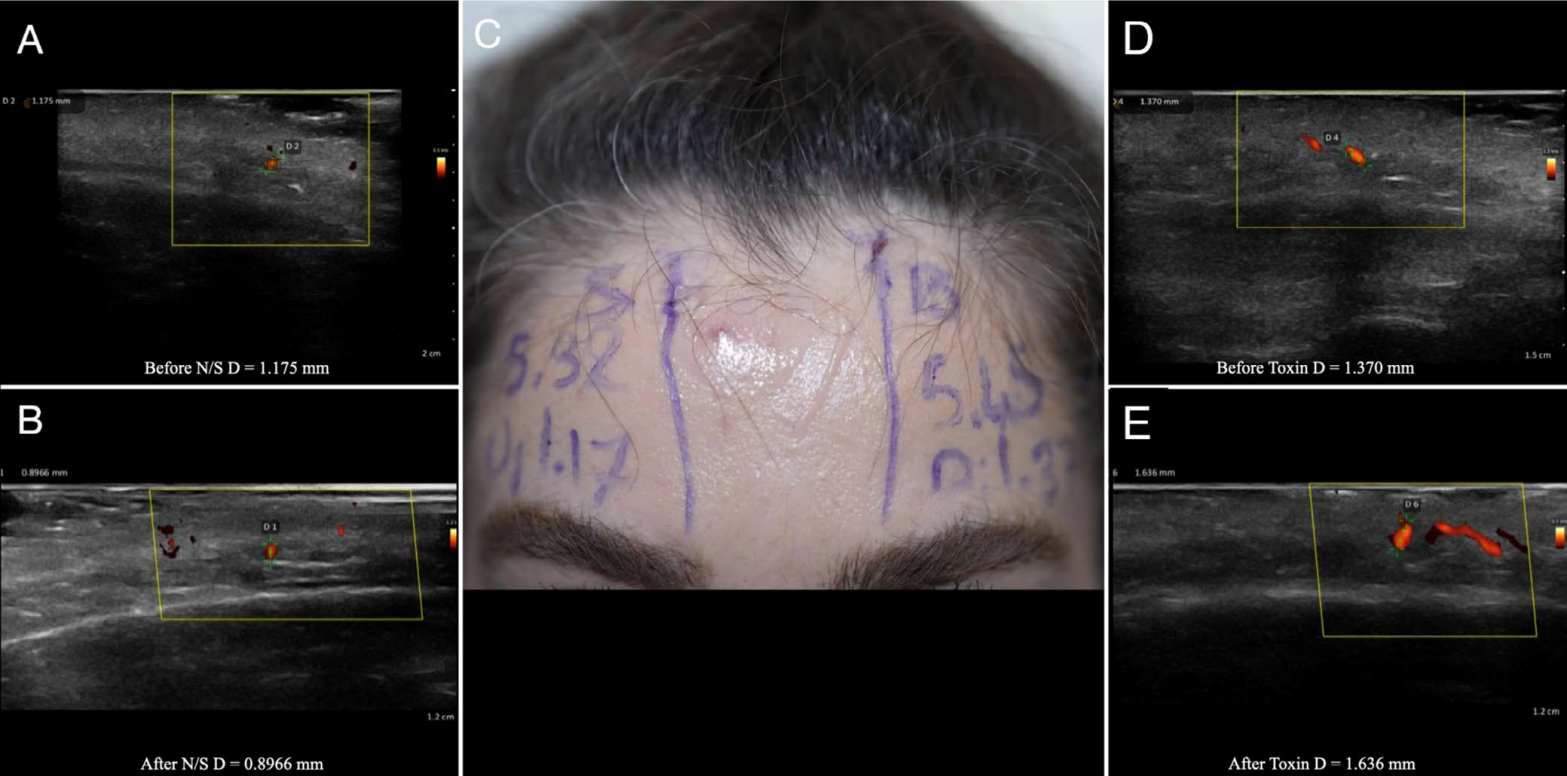
Split‐face study of a 33‐year‐old female patient, with normal saline injected into the right artery and 8 IU of botulinum toxin injected into the left supratrochlear artery. (A) Ultrasound image of the right supratrochlear artery before injection. (B) Ultrasound image of the right supratrochlear artery after injection of normal saline. (C) Marked supratrochlear arteries on the split face of the patient, with accompanying measurements. (D) Ultrasound image of the left supratrochlear artery before injection. (E) Ultrasound image of the left supratrochlear artery after injection of botulinum toxin.

**FIGURE 6:**
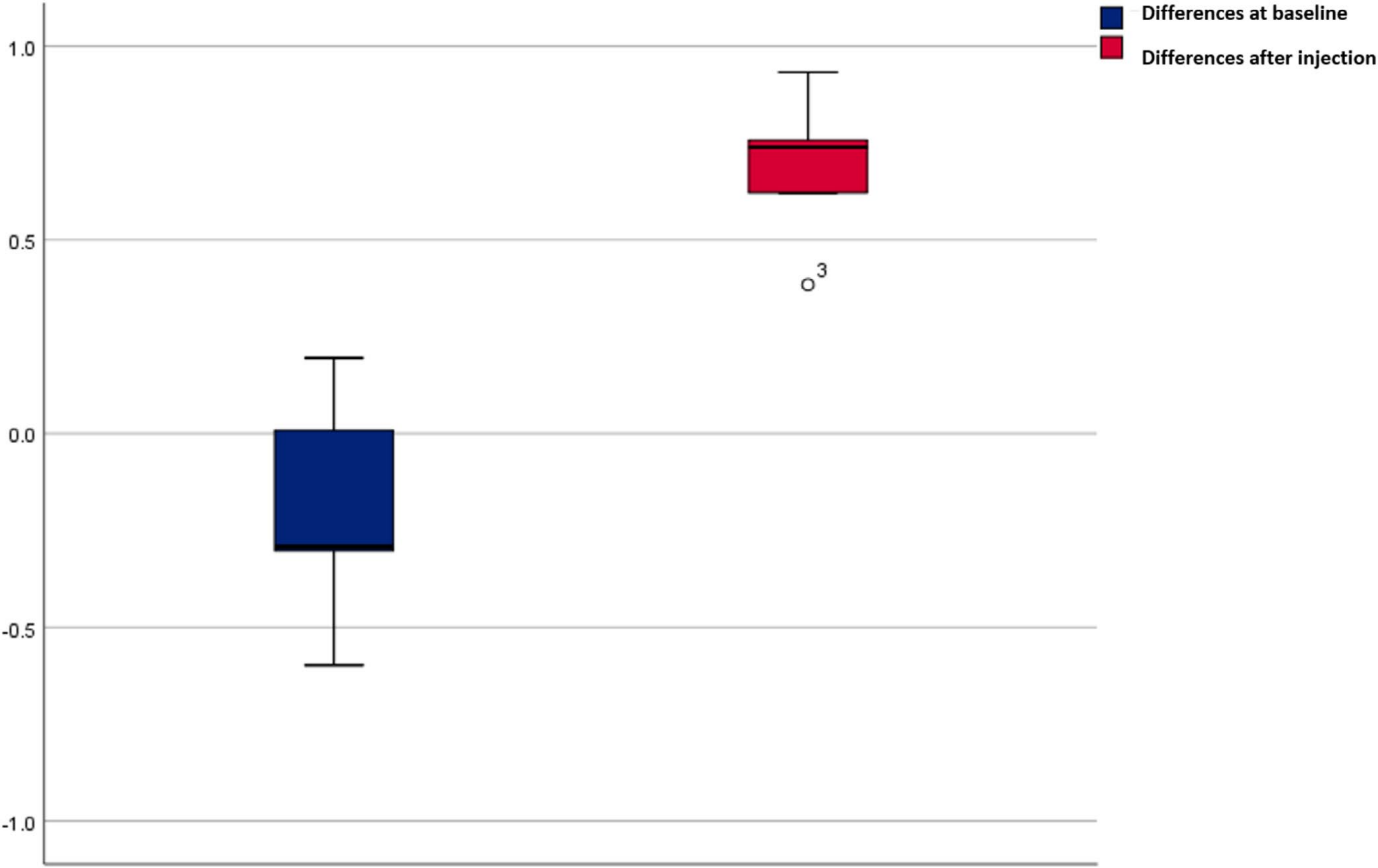
Comparison of supratrochlear artery diameter between the BoNT‐A and normal saline sides at baseline and postinjection. The *y*‐axis represents differences in the supratrochlear artery diameter (mm) for the BoNT‐A and normal saline sides, with the bars indicating the average difference.

**FIGURE 7:**
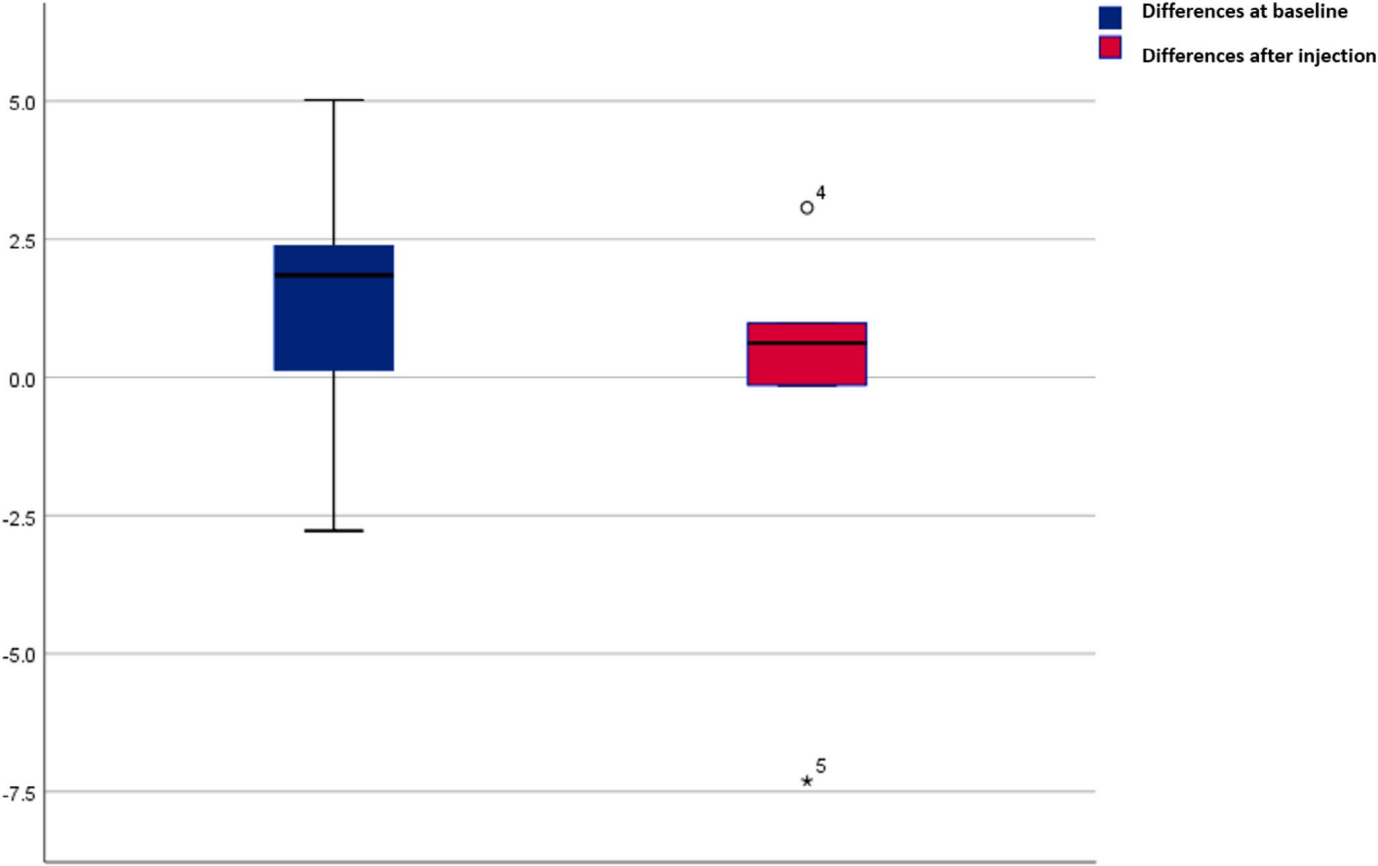
Comparison of supratrochlear artery velocity between the BoNT and normal saline sides at baseline and postinjection. The *y*‐axis represents differences in the supratrochlear artery velocity (cm/s) for the BoNT‐A and normal saline sides, with the bars indicating the average difference.

Regarding arterial velocity, the supratrochlear artery measured 6.32 ± 3.23 cm/s prior to BoNT‐A injection and decreased to 3.97 ± 1.94 cm/s afterward. For the saline control, the velocity was 5.01 ± 2.17 cm/s before and 4.53 ± 3.42 cm/s after administration (Table 1). Neither BoNT‐A (*p* = 0.34) nor normal saline (*p* = 0.68) produced a statistically significant change in arterial velocity (Figures 1, 2, 3, 4, 5). The supratrochlear artery velocity difference between the BoNT‐A and normal saline sides, both at baseline and postinjection, was 1.88 ± 4.96 mm (Figures 6 and 7).

**TABLE 1:** Clinical characteristics and arterial measurements of patients treated with botulinum toxin (Dyston 500 IU/vial + 2.5 cc normal saline, dose = 8 IU) compared to those treated with saline solution (0.9% sodium chloride, dose = 0.04 cc).

Row	Patient initial	Age	Sex	Toxin injection history	Last toxin injection (time)	Supratrochlear artery							
						Dyston (500 IU/vial + 2.5 cc N/S)/injection dose: 8 IU Abo				Normal saline (0.9 w/v sodium chloride)/injection dose: 0.04 cc			
						Arterial diameter/toxin injection		Arterial velocity/toxin injection		Arterial diameter/N/S injection		Arterial velocity/N/S injection	
						Before (mm)	After (mm)	Before (cm/s)	After (cm/s)	Before (mm)	After (mm)	Before (cm/s)	After (cm/s)
1	N.N.	25	Male	Negative	—	0.4898	1.551	5.56	3.58	0.782	0.7944	3.71	2.6
2	E.H.	26	Female	Positive	6 months ago	0.5015	1.383	12	3.57	0.8045	0.7617	6.98	2.95
3	H.A.	42	Male	Positive	6 months ago	0.9395	1.793	4.37	2.21	1.537	1.409	1.99	2.35
4	S.A.	31	Female	Positive	6 months ago	1.045	2.105	4.23	7.3	1.037	1.173	7.01	4.23
5	F.B.	33	Female	Positive	6 months ago	1.37	1.636	5.45	3.19	1.175	0.8966	5.32	10.5
6	Dyston (500 IU/vial + 2.5 cc N/S)/injection dose: 8 IU Abo = normal saline (0.9 w/v sodium chloride)/injection dose: 0.04 cc = two line of 1 cc insulin syringe												

Abbreviations: IU, international units; N/S, normal saline.

## 4 Discussion

The various applications of BoNT have gained popularity over the past two decades following the United States Food and Drug Administration's approval for treating neurological disorders. BoNT injections are now commonly used for cosmetic and medical indications, such as facial wrinkle reduction, calf and neckline contouring, focal hyperhidrosis, and treating keloids and hypertrophic scars [7].

BoNT functions through multiple pharmacological pathways, offering diverse therapeutic possibilities. At low doses, BoNT inhibits acetylcholine‐mediated vasodilation and sweat gland secretion, which is beneficial in treating hyperhidrosis [7]. At higher doses, it suppresses sympathetic‐induced vasoconstriction. Additionally, BoNT reduces the exocytosis of vasoconstrictive agents such as endothelin‐1 and von Willebrand factor, leading to vasodilation [8]. BoNT‐A specifically enhances endothelial nitric oxide synthase (eNOS) activity, cyclic guanosine monophosphate (cGMP), and soluble guanylyl cyclase (sGC) protein levels, while simultaneously suppressing arterial vasoconstriction. It also modulates smooth muscle calcium sensitization through the eNOS/sGC/cGMP pathway, contributing to arterial vasodilation [9]. Furthermore, another proposed mechanism for BoNT‐A‐induced vasodilation is the increased expression of CD31 and iNOS, which promotes endothelial cell proliferation and vasodilation [7]. These mechanisms collectively explain the vasodilatory effects of BoNT‐A in the vascular system. Future studies will further clarify the precise pathways responsible for the vasodilatory characteristics of BoNT‐A.

Given these unique characteristics, BoNT is emerging as a therapeutic option for RP. Multiple studies have demonstrated its efficacy in treating RP and digital ulcers in systemic sclerosis patients [10]. A recent meta‐analysis revealed that BoNT application in primary and secondary RP resulted in an 81.95% reduction in pain and a 79.37% improvement in digital ulcers [11].

BoNT administration has also shown promise in enhancing flap tissue survival, improving blood flow to flaps, and increasing vascular endothelial growth factor expression in animal models [12]. Preoperative BoNT injections have also improved donor site scar formation following forehead flap nasal reconstruction [13]. BoNT application to the face and neck has been associated with improved scar quality and enhanced wound healing [14]. Furthermore, BoNT‐A has demonstrated potential in treating various types of alopecia by promoting hair growth and reducing hair loss through mechanisms such as prolonging the anagen phase, reducing proinflammatory cytokines, relaxing muscles, and increasing blood flow [15].

BoNT has been shown to provide rapid pain relief within 20 min in ischemic conditions, with effects lasting for several months. As a result, it is recommended as both a first‐line and salvage therapy for vasospasm [16]. BoNT injections have also been reported to increase digital salvage and reduce pain in acute traumatic vascular hand injuries [17]. Xu and Lin [18] documented a case of chronic limb‐threatening ischemia treated with BoNT, which resulted in significant relief of rest pain. Stoehr et al. [19] presented a case of symmetric peripheral gangrene that resolved completely after BoNT injection, hypothesizing that BoNT reduces the secretion of vasoconstrictive factors and improves skin ischemia when locally administered.

Afshani et al. conducted a study comparing the safety and efficacy of Dyston (a biosimilar of abobotulinum toxin A) and Dysport (Ipsen Pharma, Switzerland) for the treatment of moderate‐to‐severe glabellar lines. Among 126 randomized participants, response rates at Day 30 were similar, with 75.44% for Dyston and 76.67% for Dysport, demonstrating the noninferiority of Dyston [20].

In our evaluation of BoNT's vasodilatory effects at higher doses, we measured the diameter of the supratrochlear arteries in patients receiving BoNT injections. Notably, the diameter of the supratrochlear artery increased significantly after BoNT administration, whereas the contralateral artery showed no change following normal saline injection. This finding may underscore the vasodilatory properties of BoNT. Based on the authors' experience, these findings are significant due to our expertise in using BoNT to manage ischemia. Additionally, we believe that administering BoNT prior to filler injections may pose a risk for intravascular events and vascular obstruction, owing to the vasodilatory effects of BoNT.

Furthermore, the baseline diameter of the left supratrochlear artery was 0.87 ± 0.38 mm, smaller than that of the right side (1.07 ± 0.31 mm), which was not significant, possibly due to side dominance in the human body. It could serve as a confounding factor affecting our data. Hafezi et al. [21] previously reported left‐sided dominance in the nose, face, and body. In addition, right‐handed individuals exhibited a larger diameter of the left vertebral artery compared to the right side, while left‐handed individuals had a greater diameter of the left internal carotid artery [22]. However, several studies have reported no side dominance for the supratrochlear artery [23]. Moreover, the depth of the supratrochlear artery was found to be similar across different facial sides, genders, and age groups [24]. The potential influence of factors such as facial side, gender, age, and other variables like BMI on arterial diameters should be further investigated in future studies to minimize potential confounding factors.

The primary limitation of this study is its small sample size. The relatively small cohort, with fewer than 25–30 patients, positions our findings as preliminary. Larger studies are necessary to validate these initial observations and to more comprehensively assess the effects of BoNT on vascular diameters following injections. Additionally, future research should explore other BoNT formulations and dosages available on the market, as the one used in this study is known for its high diffusion capacity [25], which may influence possible outcomes. Moreover, we assessed vasodilation only 30 min after BoNT‐A injection. Further studies are needed to evaluate the long‐term vasodilatory effects of BoNT and confirm these preliminary findings. Understanding BoNT‐induced vasodilation following aesthetic injections could help explain its efficacy in various ischemic conditions. Moreover, other confounding factors such as side dominance, gender, and age should be considered, as they may influence the vasodilatory effects of BoNT. Future studies should account for these variables when designing investigations into the immediate effects of BoNT on vasodilation.

## 5 Conclusion

BoNT‐A may induce vasodilation of the supratrochlear artery when injected intramuscularly, highlighting its potential utility in treating ischemic conditions. These findings are based on preliminary data, and further studies with larger sample sizes are needed to validate these results and explore the effects on other vascular territories. This vasodilatory property may provide insight into the effectiveness of BoNT in various ischemic and vasospastic conditions, expanding its therapeutic indications beyond cosmetic applications. However, additional clinical evidence is required to substantiate the use of BoNT‐A in the aforementioned conditions.

## Author Contributions

S.N., N.H., F.B., M.R.P., and N.F.‐G. conceptualized and designed the methodology for this study. S.N., N.H., F.B., M.R.P., and N.F.‐G. undertook the study execution. S.N., M.R.P., C.M.‐G., and N.F.‐G. prepared the original draft. All authors contributed to the critical review, commentary, and revision of the original manuscript.

## Disclosure

Data Policy: For this type of study, we do not have data to deposit in a public repository.

## Ethics Statement

All treatments were performed in adherence to the Declaration of Helsinki and in accordance with the standards of good clinical care following local guidelines and regulations. This article does not contain any studies with animals performed by any of the authors.

## Consent

All patients included in this study provided written informed consent to access their patient charts and extract their data for the purposes of this study. No charts were accessed if patients declined their participation in this study. All participants have provided consent for the publication of their photographs.

## Conflicts of Interest

The authors declare no conflicts of interest.

## Data Availability

The data that support the findings of this study are available from the corresponding author upon reasonable request.
